# Protective Effect of Placental Mesenchymal Stromal Cells in an In Vitro Model of Parkinson’s Disease Using Differentiated Neuroblastoma Cells

**DOI:** 10.3390/ijms27093925

**Published:** 2026-04-28

**Authors:** Almudena Coto-Vilcapoma, Laura Sánchez-Carretero, Daniel Arenas-Gonzalez, José A. Molina, María José Morán-Jiménez, José Joaquín Merino, Paz de la Torre, Ana I. Flores

**Affiliations:** 1 Grupo de Medicina Regenerativa, Instituto de Investigación Sanitaria Hospital 12 de Octubre (Imas12), 28041 Madrid, Spain; almudena.coto.sb@gmail.com (A.C.-V.); laurasanchez.imas12@h12o.es (L.S.-C.); dani.arenas.imas12@h12o.es (D.A.-G.); moranjimenez@h12o.es (M.J.M.-J.); josejmerino.imas12@h12o.es (J.J.M.); torre-merino.paz@h12o.es (P.d.l.T.); 2 Servicio de Neurología e Instituto de Investigación Hospital 12 de Octubre (Imas12), 28041 Madrid, Spain; cvillaiza@telefonica.net; 3 Departamento de Farmacología, Farmacognosia y Botánica, Facultad de Farmacia, Universidad Complutense de Madrid (UCM), 28040 Madrid, Spain

**Keywords:** perinatal mesenchymal stromal cells, perinatal MSCs, decidua mesenchymal stromal cells, DMSCs, dibutyryl cyclic adenosine monophosphate, dbcAMP, 1-methyl-4-phenylpyridinium, MPP+, NB69, Parkinson, PD, neuroprotection, oxidative stress

## Abstract

Parkinson’s disease (PD) is the second most prevalent neurodegenerative disorder. It is characterized by the accumulation of misfolded α-synuclein (α-syn) and progressive loss of dopaminergic neurons in the substantia nigra. Due to the limitations of current therapies, mesenchymal stromal cell (MSC) transplantation has emerged as a promising neuroprotective strategy. This study evaluated the neuroprotective potential of decidua-derived mesenchymal stromal cells (DMSCs) in vitro using a human neuroblastoma cell line (NB69) exposed to the neurotoxin 1-methyl-4-phenylpyridinium (MPP+) as a PD model. The NB69 cells were differentiated into a mature dopaminergic phenotype using dibutyryl cyclic adenosine monophosphate (dbcAMP) and then exposed to MPP+. In proliferative NB69 cells, the effect of DMSCs was masked by their inherent antitumor activity against the neuroblastoma phenotype. Conversely, in the differentiated NB69 model, DMSCs demonstrated a significant protective role against MPP+-induced cytotoxicity. Interestingly, the mechanism by which DMSCs might exert a neuroprotective effect against MPP+ damage in differentiated NB69 cells appears to involve improving mitochondrial function by reducing free radicals. In summary, these findings suggest that DMSCs exert a neuroprotective effect in a dopaminergic-like context and highlight the importance of using differentiated cell models to accurately evaluate cell-based therapies for PD in the striatum.

## 1. Introduction

Parkinson’s disease (PD) is the second most common neurodegenerative disorder, affecting 1% of people over 60 years of age [1]. Between 5 and 10% of PD cases have a genetic origin, resulting in early-onset PD. However, the majority of PD cases are idiopathic and are associated with aging. In addition to genetic predisposition, other risk factors include environmental toxins, pesticides, heavy metals, traumatic injuries and bacterial or viral infections [2]. These factors produce an inflammatory process that contributes to the development and progression of parkinsonian symptoms.

PD is characterized by the accumulation of misfolded α-synuclein (α-syn), which results in the progressive loss of dopamine-secreting neurons in the substantia nigra and motor impairment [3]. This manifests mainly as bradykinesia, postural instability, rigidity and resting tremor [4]. Additionally, the PD patients present with non-motor symptoms, such as mood disorders and cognitive dysfunction [5].

The presence of Lewy bodies has been determined in several regions of the nervous system, including dopaminergic and non-dopaminergic, which could contribute to PD symptoms [6]. Lewy bodies are insoluble cytoplasmic aggregates containing fibrillar α-syn [7], and the loss of functional α-syn affects dopamine homeostasis [8]. Furthermore, it has been proposed that α-syn may inhibit mitochondrial complex I, resulting in the mitochondrial dysfunction observed in many patients [9], as well as the systemic brain inflammation and oxidative stress responses involved in the PD pathogenesis [10,11].

One of the most studied toxins is 1-methyl-4-phenyl-1,2,3,6-tetrahydropyridine (MPTP). MPTP induces symptoms similar to those of PD and has been widely used as a PD model in experimental animals and in neuroblastoma cell lines [6,12]. MPTP crosses the blood–brain barrier, showing high selectivity for the nigrostriatal dopaminergic system. It can enter cells via the dopamine transporter after being oxidized by monoamine oxidase B (MAO-B) into 1-methyl-4-phenylpyridinium (MPP+) [13]. MPP+ is a useful model for studying the effects of reactive oxygen species (ROS) generation and is used to evaluate the antioxidant effects of potential therapeutic agents [14,15]. In the human neuroblastoma cell line SH-SY5Y, MPP+ phosphorylates extracellular signal-regulated kinases (ERK), while decreasing p38 phosphorylation and c-Jun N-terminal kinases (JNK), as a neurotoxic mechanism of MPP+ treatment [16,17]. Inside the cell, MPP+ targets mitochondria. At high concentrations, it partially inhibits mitochondrial complex I activity, resulting in mitochondrial depolarization and increased oxidative stress [18,19]. These toxic consequences, together with other, as yet unexplained, effects on energy metabolism, promote the dopaminergic neuronal death observed in the parkinsonian syndrome caused by MPTP.

Currently, there is no cure for PD, though several therapeutic strategies exist to treat motor symptoms [20]. However, these treatments primarily address motor symptoms with little to no impact on non-motor symptoms or disease progression [21]. Levodopa is the most effective drug for managing PD motor symptoms (e.g., rigidity, slowness, and tremor) [22], but its long-term use causes motor complications in most patients [23]. Therefore, studying and developing new therapies is necessary, and cell-based therapies have emerged as a promising approach to treating PD.

Stem cell therapy may be a viable treatment option for PD. This therapy involves replacing and/or repairing damaged dopamine-producing nerve cells through an intracerebral transplant. Several clinical trials have been conducted using different types of stem cells, including embryonic stem cells (ESCs), induced pluripotent stem cells (iPSCs) and fetal neural stem cells that have been differentiated into dopaminergic neurons [24,25]. These cells engraft into the host tissue and provide an improvement in PD symptoms [26,27]. However, the expected results have not been achieved. For example, clinical trials such as the one carried out by Piccini, P., et al. [28] have shown that the efficacy achieved depends on the affected area [29]. Furthermore, negative effects have been observed, including the spread of the pathology to the cell graft and an increase in non-motor symptoms. This has driven the search for alternative strategies.

Some of these strategies are based on transplanting dopaminergic neurons derived from other stem cell sources [27], such as mesenchymal stromal cells (MSCs). Once transplanted into damaged tissue, MSCs have been shown to restore dopaminergic connections to the striatum and areas it supplies, thereby reversing motor deficits [30]. Furthermore, MSCs exhibit low immunogenicity and a low risk of forming teratomas, making them promising candidates for cell therapies [31]. Once transplanted, MSCs can migrate to the site of damage and perform numerous paracrine functions that can result in tissue regeneration and symptom improvement [32,33]. Their most relevant functions include neuroprotection, induction of neurogenesis, immunomodulation, and prevention of protein misfolding [34]. This wide range of functions indicates that MSCs have the potential to mitigate various pathological factors in neurodegenerative diseases.

MSCs can be obtained from a wide variety of neonatal and adult tissues, including bone marrow, which is already used to treat this pathology [34]. The placenta is an alternative source for obtaining MSCs without an invasive isolation procedure, and the cells have several advantages over those obtained from other sources [35,36]. Decidua mesenchymal stromal cells (DMSCs) originate from the maternal side of the placenta and exhibit intermediate characteristics between embryonic cells and adult stem cells [36]. Furthermore, DMSCs have low risk of viral infection. Like other MSCs, they are hypoimmunogenic and lack teratogenic potential, which justifies their use in cell therapy [36]. In vitro and in vivo studies have demonstrated DMSCs’ ability to migrate to damaged tissues. This suggests that DMSCs could serve as therapeutic agents in cell therapy-based treatments and as cellular vehicles for therapeutic agents [33,37,38,39,40,41,42,43]. DMSCs modulate inflammation by suppressing pro-inflammatory immune responses and promoting anti-inflammatory pathways, largely by regulating immune cells and secreting immunomodulatory factors [37,39].

The first objective of this study was to evaluate whether the NB69 human neuroblastoma cell line, when exposed to the neurotoxin MPP+, could serve as an in vitro model of dopaminergic (DA) neurons for PD research and to compare it with differentiated NB69 cells. Having established this, the main objective was to study the potential neuroprotective role of DMSCs against MPP+-induced neurotoxicity in the NB69 neuroblastoma cell line for the treatment of PD.

## 2. Results

### 2.1. 1-Methyl-4-Phenylpyridinium (MPP+) Induces Cell Death by Apoptosis in NB69 Neuroblastoma Cells

**Figure 1:**
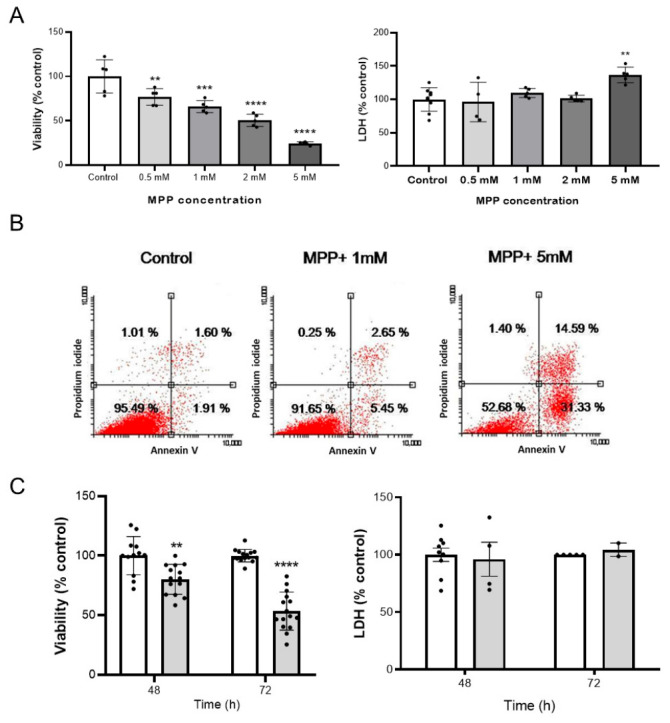
Effects of 1-methyl-4-phenylpyridinium (MPP+) on NB69 neuroblastoma cells. (**A**) Percentages of cell viability (*n* = 5 independent experiments) and cytotoxicity (lactate dehydrogenase (LDH) release, *n* ≥ 4 independent experiments) treated with the neurotoxin MPP+ at doses of 0.5, 1, 2, or 5 mM for 48 h and relative to the untreated NB69 cell line (control). After 48 h, MPP+ treatment caused a significant, dose-dependent reduction in NB69 cell viability without inducing cell death. (**B**) Flow cytometry evaluation of cell viability and type of cell death (apoptosis versus necrosis) of untreated (control) and treated NB69 cells with doses of 1 or 5 mM of the neurotoxin MPP+ for 48 h. Cells were stained with annexin V, a marker of apoptosis, and with propidium iodide, a marker of necrosis or loss of membrane integrity. Untreated NB69 cells (control) and low doses of MPP+ showed no evidence of apoptosis, which is consistent with the absence of LDH release at concentrations below 5 mM. (**C**) Cell viability (*n* ≥ 4 independent experiments) and LDH concentration (*n* ≥ 2 independent experiments) (% relative to control) of the NB69 cell line treated with the neurotoxin MPP+ at 0.5 mM for 48 or 72 h. Time-course analysis of the 0.5 mM dose showed a significantly greater reduction in NB69 viability at 72 h compared with 48 h, without an increase in cell death. The number of replicates per experiment varied. Data are represented as mean ± SD. ** *p* < 0.01, *** *p* < 0.001, **** *p* < 0.0001 vs. control.

1-methyl-4-phenylpyridinium (MPP+), the active neurotoxic metabolite of 1-methyl-4-phenyl-1,2,3,6-tetrahydropyridine (MPTP), has been shown to induce neurotoxicity in dopaminergic neurons, resulting in a syndrome that is clinically and pathologically similar to Parkinson’s disease (PD). First, we studied the effects of MPP+ on NB69 neuroblastoma cells. Treatment with MPP+ resulted in a significant loss of cell viability in NB69 cells after 48 h, as determined by the Alamar blue test. This effect was dose-dependent, increasing significantly with the dose (F (4, 20) = 37.72, *p* < 0.0001) (Figure 1A). At the lowest studied dose, 0.5 mM, around 20% loss of NB69 viability was observed; this increased to 75% at a 5 mM dose. This loss of NB69 viability could be a consequence of cytotoxicity, decreased cell proliferation, and decreased cellular metabolic activity. The activity of cytoplasmic enzymes released into the medium by damaged cells is a biomarker of cellular cytotoxicity. Traditionally, lactate dehydrogenase (LDH) release is associated with necrosis, though it can also occur in the late stages of apoptosis or other forms of lytic cell death. LDH release was only significant at the 5 mM dose, with a 30% increase compared to the control (*p* < 0.001; Figure 1A). To detect early apoptosis, we measured phosphatidylserine (PS) on the cell membrane by flow cytometry using fluorescently labeled annexin V. Propidium iodide (PI), a live cell membrane-impermeable fluorescent dye, was used to detect dead cells by flow cytometry. Flow cytometry analysis revealed that cells treated with 5 mM of MPP+ lost viability and showed signs of early (Annexin V+ PI−) and late (Annexin V+ PI+) apoptosis at this dose, which correlated with positivity in LDH release. At this highest dose, almost 46% of the cells were apoptotic: 31.33% showed signs of early apoptosis, and 14.59% showed signs of late apoptosis (Figure 1B). Untreated NB69 samples (control) showed no signs of apoptosis (Annexin V− PI−, Figure 1B), with 95.49% of the cells being viable. At a dose of 1 mM MPP+, the cells exhibited 5.45% and 2.65% of early and late apoptosis, respectively. This correlated with the negativity of LDH release observed at doses lower than 5 mM.

Since we needed to induce cellular damage in NB69 cells without causing irreversible damage, we decided to use a dose below 5 mM. We selected the lowest tested dose, 0.5 mM, at which we observed a significant loss of viability without signs of cytotoxicity (Figure 1A). Studying the effect of the 0.5 mM dose over time revealed a significantly greater negative impact on NB69 viability when exposure time increased from 48 to 72 h, resulting in a 50% decrease in viability compared to the control (Figure 1C). However, no increase in cell death was observed, as measured by LDH release (Figure 1C). Based on these results, the experiments in this study were performed with a dose of 0.5 mM and a treatment duration of 72 h.

### 2.2. Analysis of the Possible Antitumor Effect of Decidua-Derived Mesenchymal Stromal Cells (DMSCs) on NB69 Neuroblastoma Cells

**Figure 2:**
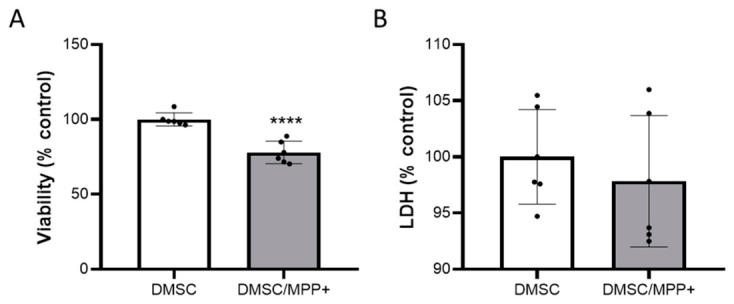
Effect of 0.5mM of the neurotoxin MPP+ on DMSC viability after 72 h of treatment. (**A**) DMSC cell viability and (**B**) DMSC cell death measured as LDH release into the culture medium. Values are represented as mean ± SD and calculated as a percentage relative to untreated cells (% control) (*n* = 3 independent experiments, each with two replicates). MPP+ exerted a significant negative effect on DMSC viability without inducing cell death under the conditions tested. **** *p* < 0.0001 vs. control.

The objective of this study was to evaluate the protective effect of decidua-derived mesenchymal stromal cells (DMSCs) on NB69 cells in a toxicity model. First, it was necessary to determine whether the neurotoxin could affect DMSCs. At a dose of 0.5 mM, MPP+ was observed to have a significantly negative effect on DMSC viability, with a loss of nearly 20% after 72 h. However, no LDH release was observed, suggesting that the neurotoxin does not induce DMSC cell death at this dose and time interval (Figure 2).

**Figure 3:**
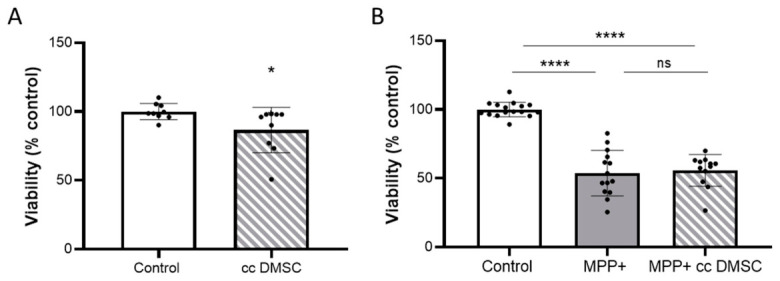
Effects of DMSCs on NB69 neuroblastoma cells with and without MPP+ treatment. (**A**) Cell viability (% relative to control) of the NB69 cell line in the presence of DMSCs at a 1:5 ratio after 72 h of co-culture (cc) (*n* = 3 independent experiments, each performed in triplicate). NB69 cell viability was negatively affected after 72 h of co-culture with DMSCs. (**B**) Cell viability (% relative to control) of the NB69 cell line treated with 0.5 mM MPP+ and co-cultured without and with DMSCs for 72 h (*n* ≥ 4 independent experiments, each performed in triplicate). DMSCs showed no protective effect on NB69 cells exposed to MPP+ (ns). Data are represented as mean ± SD. * *p* < 0.05, **** *p* < 0.0001 vs. control.

We have previously demonstrated that DMSCs negatively affect the viability of tumor cells [33]. Therefore, given that NB69 cells are a neuroblastoma cell line, we first studied the effect of DMSCs on their viability. We conducted these experiments using the Transwell co-culture system, in which the two cell types do not come into contact, and only signaling molecules are permitted to pass through. We observed that DMSCs negatively affected NB69 viability when co-cultured at a 1:5 ratio (DMSC:NB69) after 72 h (Figure 3A). This negative effect was statistically significant (*p* < 0.05). These results are consistent with the hypothesis that DMSCs can probably recognize cellular stress signals from NB69 tumor cells and respond by inducing cellular death. Furthermore, we observed that this effect depended on the ratio between DMSCs and NB69. It was also significant (*p* < 0.05) at the 1:2 ratio (DMSC:NB69) but not at a 1:10 ratio, where only a slight decrease in viability was observed. All subsequent co-culture experiments were performed at a 1:5 DMSC:NB69 ratio.

In this context, the NB69-based in vitro PD model was not suitable for investigating the protective role of DMSCs in our study. Nevertheless, we performed co-cultures of DMSCs with NB69 cells treated with MPP+ (Figure 3B). As expected, treatment with MPP+ significantly reduced the viability of NB69 cells, whether cultured alone or with DMSCs (F (2, 39) = 72.74, *p* < 0.0001). Additionally, the results showed that the DMSCs did not protect the NB69 cells from MPP+-induced damage (Figure 3B). These results led us to rule out the use of undifferentiated NB69 cells as a model and prompted us to consider differentiated NB69 instead.

### 2.3. Differentiation of the Human Neuroblastoma Cell Line NB69 into Dopaminergic Neurons by Dibutyryl Cyclic Adenosine Monophosphate (dbcAMP) Treatment

**Figure 4:**
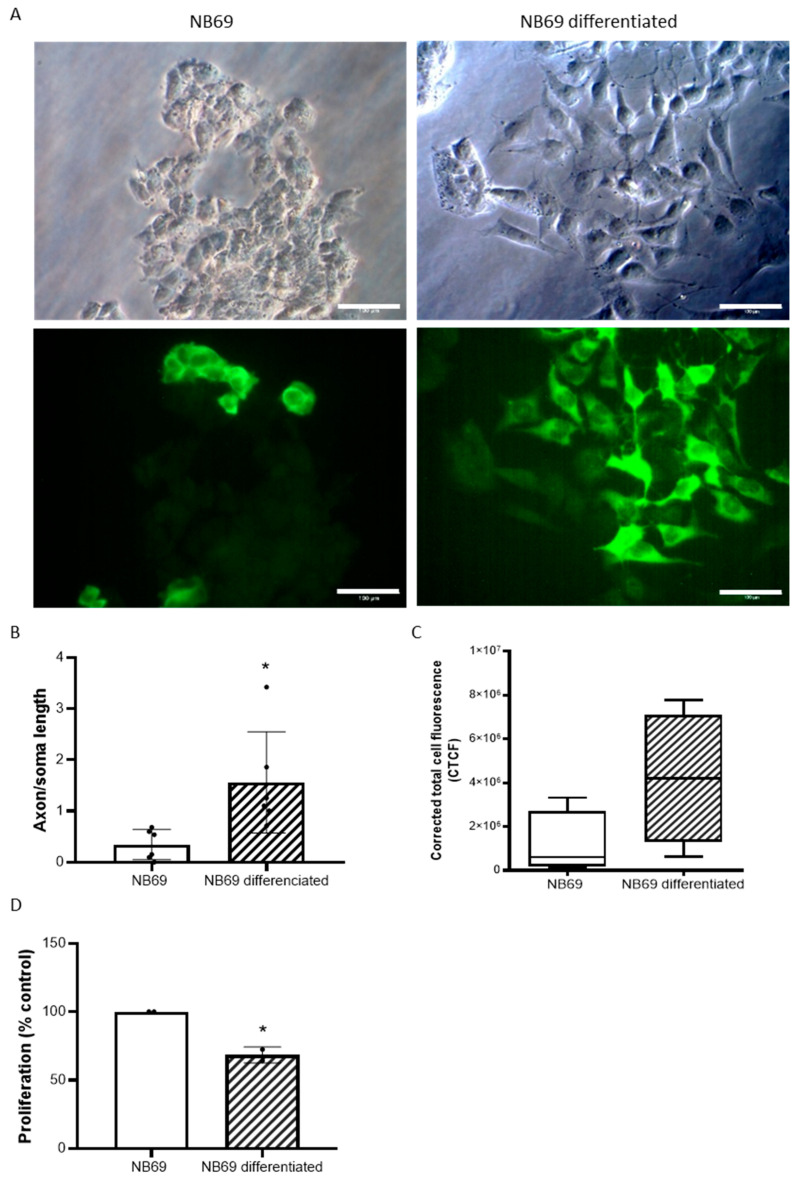
Differentiation of NB69 neuroblastoma cells into dopaminergic neurons. (**A**) Bright-field and fluorescence microscopy of TH-positive cells of the NB69 cell line (**left**) or NB69 cells differentiated with 2 mM dbcAMP for 5 days (**right**). Differentiation with dibutyryl-cAMP (dbcAMP) induced significant morphological changes in NB69 cells, characterized by polarization, neurite extension, and branching. (**B**) Relationship between axon length and soma length of NB69 cells after 7 days in culture with and without the differentiation compound dbcAMP, quantified in 5 fields of view. NB69 cells differentiated with dbcAMP show a significantly increased relationship between axon length and soma size compared with undifferentiated cells. (**C**) Fluorescence intensity for TH in NB69 cells and differentiated NB69 cells with 2 mM dbcAMP, quantified in 4 fields of view. Differentiation with dbcAMP resulted in biochemical alterations in NB69 cells, evidenced by increased TH-positive cell numbers and higher TH protein levels. (**D**) Cell proliferation rate of differentiated NB69 relative to control (*n* = 2 independent experiments). NB69 cell differentiation was further confirmed by a significant reduction in proliferation rate. Data is represented as mean ± SD. * *p* < 0.05 vs. control. Scale bar = 100 μm.

To study the effects of DMSCs on differentiated NB69 cells, we treated the cells with dibutyryl cyclic adenosine monophosphate (dbcAMP). Exposure to a 5-day treatment of dbcAMP has been shown to induce changes in the NB69 cell line, including increased tyrosine hydroxylase (TH) activity and decreased proliferation, which are compatible with a shift toward a dopaminergic-like phenotype [44]. We differentiated human neuroblastoma NB69 cells by adding dbcAMP to the culture medium. As expected, NB69 cells acquired significant morphological and biochemical changes as a consequence of differentiation (Figure 4). Morphological modifications included extensive neurite growth; thus, differentiated NB69 exhibited a more neuron-like morphology compared to NB69 cells (*p* < 0.05; Figure 4A). We also observed that the cell body became polarized and that the neurites extended and branched (Figure 4B). Additionally, differentiated NB69 cells appeared to extend neurite-like processes toward neighboring cells, suggesting the formation of intercellular connections. The biochemical changes consisted of increased immunofluorescence levels of the TH protein due to an increased number of TH-positive cells following differentiation by dbcAMP, as well as higher levels of TH protein expression within the cells (Figure 4C). Furthermore, NB69 cell differentiation was confirmed by a significant decrease in the proliferation rate (Figure 4D).

### 2.4. Effect of Neurotoxin MPP+ on Differentiated NB69 Cells in the Presence or Absence of DMSCs

**Figure 5:**
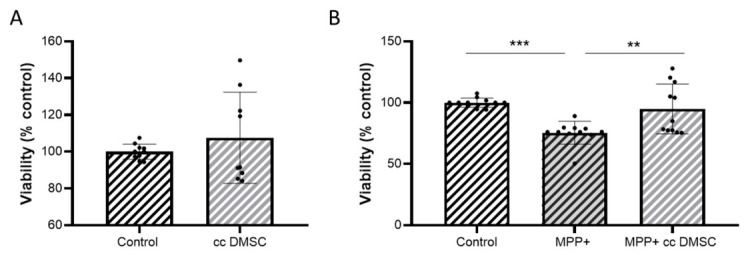
Effects of DMSCs on differentiated NB69 neuroblastoma cells with and without MPP+ treatment. (**A**) Cell viability (% relative to control, black bar) of the differentiated NB69 cell line in the presence of DMSCs in 1:5 ratios after 72 h of co-culture (cc) (*n* = 3 independent experiments, at least 2 replicates each). DMSCs did not negatively affect the viability of differentiated NB69 cells. (**B**) Cell viability (% relative to control) of the differentiated NB69 cell line treated with 0.5 mM MPP+ and then co-cultured without and with DMSCs for 72 h (*n* = 3 independent experiments, at least 3 replicates each). DMSCs treatment significantly attenuated MPP+-induced damage in differentiated NB69 cells. Data are represented as mean ± SD. ** *p* < 0.01, *** *p* < 0.001.

We previously observed that DMSCs negatively affect NB69 cell viability after 72 h of co-culture (Figure 3A). Therefore, we studied the effect of DMSC:NB69 co-culture at a 1:5 ratio on differentiated NB69 cells. Interestingly, the DMSCs did not have a negative effect on the viability of the differentiated NB69 cells (Figure 5A). This reduced susceptibility to DMSC effects is consistent with the cells displaying a more stable, neuron-like phenotype.

The effect of DMSCs on damaged, differentiated NB69 cells treated with MPP+ was studied. The results revealed that DMSCs significantly protected against MPP+-induced damage (F (2, 30) = 10.72 *p* = 0.0003) (Figure 5B). Unlike undifferentiated NB69 cells, co-culturing differentiated NB69 cells with DMSCs restored viability lost due to MMP+ treatment. This finding is significant because it demonstrates that DMSCs kill NB69 tumor cells while preserving differentiated NB69 cells, which are much more akin to TH+ dopaminergic neurons. These experiments were performed by co-culturing NB69 cells with DMSCs in a Transwell chamber, ensuring there was no direct contact between the cells. These results suggest that DMSCs’ regenerative capabilities are mediated by paracrine mechanisms, confirming the role of the factors secreted by DMSCs in neuronal regeneration.

### 2.5. Study of the In Vitro Migratory Capacity of DMSCs in the Presence of NB69 Cells and Differentiated NB69 Cells

**Figure 6:**
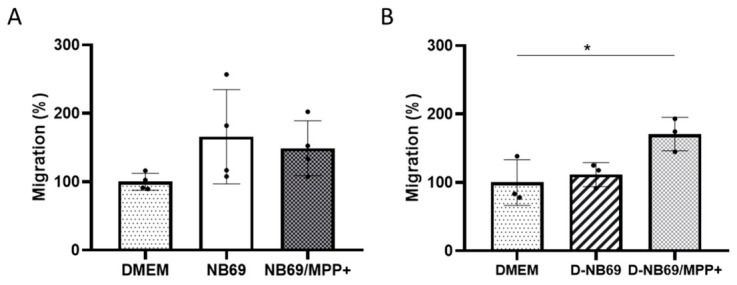
Migration of DMSCs towards malignant or differentiated NB69 cells, in the absence or presence of the toxic MPP+. Relative migration of DMSCs toward undifferentiated NB69 cells ((**A**) *n* = 6 independent experiments) or differentiated NB69 cells ((**B**) *n* = 3 independent experiments) without treatment or after treatment with 0.5 mM MPP+ for 72 h. DMSC migration was unchanged toward undifferentiated NB69 cells following MPP+ treatment (**A**) but was significantly increased toward MPP+-damaged differentiated NB69 cells (**B**). Data are calculated as a percentage with respect to control migration without cells (DMEM). Values are represented as mean ± SD. * *p* < 0.05 vs. control.

Due to the DMSCs’ migratory capacity to sites of cellular damage [33], we analyzed whether MPP+-induced toxicity could affect their migratory capacity in vitro. PD models commonly use MPTP treatment, so it is important to evaluate whether DMSCs could be administered intravenously or if local transplantation would be necessary in future in vivo neurotoxin studies [33,42]. To that end, DMSCs were co-cultured with NB69 cells that were either undifferentiated or differentiated and had been treated with or without 0.5 mM MPP+ for 72 h. The results showed that NB69 cells act as a chemoattractant for DMSCs, inducing their migration more effectively than cell-free culture medium (DMEM), though the difference was not statistically significant (Figure 6A). Interestingly, MPP+ treatment cells did not increase DMSC migration toward NB69 cells (Figure 6A). These results suggest that NB69 neuroblastoma cells secrete chemoattractants that stimulate DMSC migration. This effect likely masks the chemoattractive effect of MMP+ damage. In contrast, differentiated NB69 cells did not induce DMSC migration. However, MPP+-damaged differentiated NB69 cells significantly increased DMSC migration compared to the control (F (2, 6) = 6.443, *p* = 0.032, Figure 6B). These results demonstrate that factors present in MPP+-damaged differentiated NB69 cells significantly attract DMSCs and support the use of the intravenous route in in vivo PD studies. These factors are not present in healthy differentiated NB69 cells.

### 2.6. Study of the Mechanism by Which DMSCs Protect NB69 Cells Damaged by the Toxic MPP+

**Figure 7:**
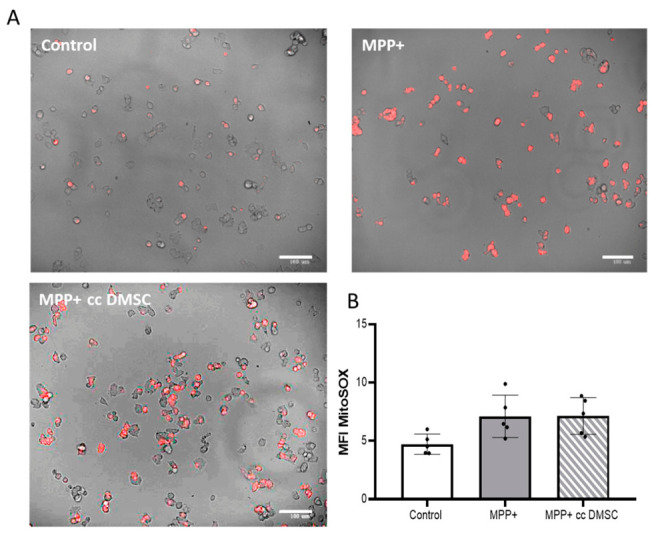
Representative fluorescence intensity profiles in untreated NB69 cells (control) and MPP+-treated cells. (**A**) Live NB69 cells were left untreated (control), treated with MPP+ for 48 h (MPP+), or treated with MPP+ and co-cultured with DMSCs for 72 h (MPP+ cc DMSC). Cells were then labeled with MitoSOX™ Red indicator and pictures were taken by a ZOE Fluorescent Cell Imager. The images are representative of each condition in which five images were captured at random. (**B**) Mean fluorescence intensity (MFI) of each positive cell after MitoSOX staining from five randomly selected representative images. MFI is a quantitative measure used to determine the levels of mitochondrial superoxide, a key ROS. An increase in MFI in MPP+-treated NB69 cells without or with co-culture with DMSCs (MPP+ and MPP+ cc DMSC, respectively) indicates increased mitochondrial superoxide production after 48 h of treatment.

We investigated the mechanisms by which DMSCs might protect neurons from MPP+ neurotoxin damage. To determine if the protective effect of DMSCs was related to improved mitochondrial function, we used a MitoSOX-based assay to detect superoxide/ROS production inside mitochondria via microscopy and flow cytometry. An accumulation of superoxide levels was observed in NB69 cells treated with 0.5 mM MPP+ for 48 h compared to the control group (Figure 7). This was due to an increase in the number of positive cells and in the intensity of their fluorescence (Figure 7A). This increase was determined quantitatively by measuring the mean fluorescence intensity (MFI) (Figure 7B). These results suggest that the neurotoxin MPP+ inhibits mitochondrial respiration and that mitochondrial dysfunction leads to high levels of reactive oxygen species (ROS). Treatment with MPP+ increased MFI in NB69 cells in both the absence and presence of DMSC co-culture (MPP+ and MPP+-cc DMSC), suggesting an increase in mitochondrial superoxide production after 48 h of treatment. However, this increase was not statistically significant (F (2, 12) = 4.431, *p* = 0.0362). Conversely, co-culture with DMSCs had no effect on the level of superoxide in MPP+-treated NB69 cells (Figure 7B). The absence of an effect of co-culture with DMSCs on superoxide production is consistent with the lack of improvement in NB69 cell viability observed with MPP+ treatment (Figure 3B). These results suggest that DMSCs are unable to effectively modulate oxidative stress in this model.

**Figure 8:**
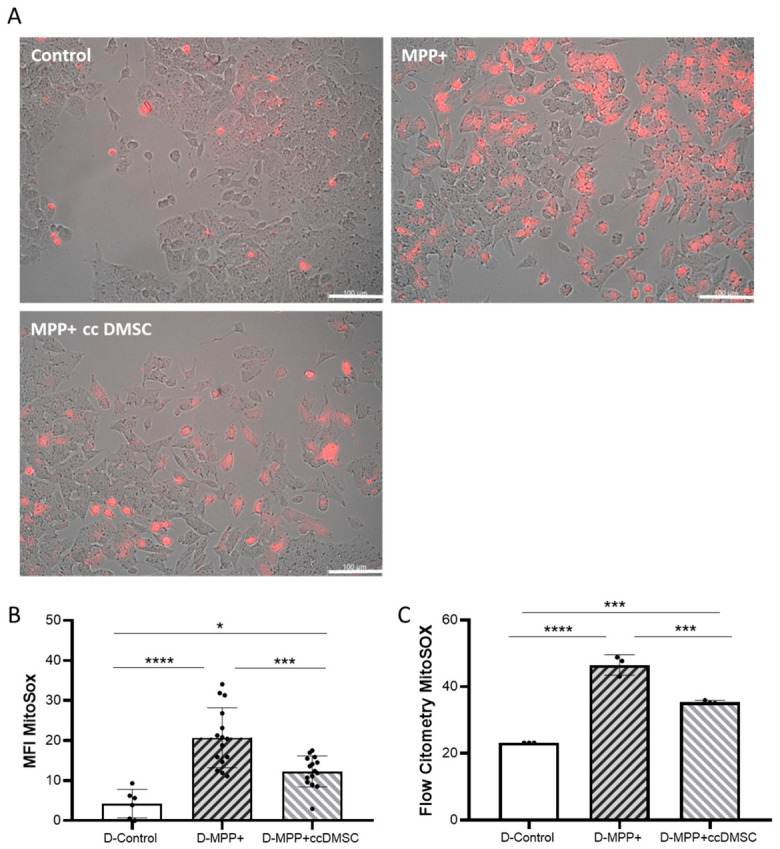
Effect of co-culture with DMSCs on mitochondrial superoxide production measured using the MitoSox probe in differentiated NB69 cells. NB69 cells were left untreated (D-Control), treated with 0.5 mM MPP+ for 48 h without co-culture (D-MPP+), or cultured with DMSCs during MPP+ treatment (D-MPP+ cc DMSC). (**A**) Representative images of each condition were taken using a fluorescence microscope (20×). (**B**) The graph shows the quantification of red fluorescence of five randomly selected images, representing the mean ± SD, analyzed via one-way ANOVA and Bonferroni test. (**C**) Quantification by flow cytometry of the percentage of cells positive to MitoSOX staining (*n* = 3 independent experiments). * *p* < 0.05; *** *p* < 0.001; **** *p* < 0.0001.

Next, we performed a MitoSox staining assay on the differentiated NB69 cell line, both with and without MMP+ treatment, and in co-culture with DMSCs. Quantification of live images revealed low baseline levels of fluorescence intensity in the differentiated NB69 cells (D-Control), indicating low ROS levels. Treatment with 0.5 mM MPP significantly increased superoxide anion levels by fivefold compared to the control group (D-MPP+; **** *p* < 0.0001). However, when co-cultured with DMSCs, ROS levels decreased significantly (D-MPP+ cc DMSC; *** *p* < 0.001). Nevertheless, they remained significantly higher than those in the control group (F (2, 33) = 20.05 *p* < 0.0001) (Figure 8A,B). We observed a similar pattern using flow cytometry (Figure 8C). In differentiated NB69 cells (D-Control), only 23% of the total population was positive for mitochondrial ROS. When these cells were treated with 0.5 mM MPP+, the ROS-positive population increased significantly to over 46% (D-MPP+; *p* < 0.001). However, when these damaged cells were co-cultured with DMSC, the percentage of positive cells decreased to 35% (D-MPP+ cc DMSC; *p* < 0.01). This suggests that DMSCs play a reparative role on damaged neuronal-like cells (F (2, 6) = 129.1, *p* < 0.0001). These results suggest that DMSCs exert a neuroprotective effect against MPP+ neurotoxin by preserving mitochondrial function.

## 3. Discussion

In vitro experimental models of Parkinson’s disease (PD) offer an alternative approach to understanding neuronal deterioration and reduce the need for laboratory animal testing. In vitro studies commonly use cell lines because they are highly reproducible. However, due to their oncogenic properties, these cells do not accurately reproduce neuronal morphology and physiology. Therefore, differentiation toward a less tumorigenic phenotype is being studied to better understand the disease [45]. In vitro PD models include cell lines, primary substantia nigra cultures, primary striatum cultures, induced pluripotent stem cells (iPSCs), and midbrain organoids [46]. Each model has its own advantages and disadvantages. However, a reliable, optimal model that can be used to understand all aspects of PD etiology is not yet available. New differentiation protocols for iPSC cells and organoids have better molecular and functional characteristics for studying neurodegenerative diseases such as PD; however, they are much more complex and difficult to manage. Therefore, it is necessary to study new models that complement this knowledge. Human neuroblastoma NB69 cells are an interesting model for studying the effects of neurotoxic and neuroprotective drugs on catecholaminergic neurons [44,47,48]. The NB69 cell line is rich in catecholamines and has a suitable biochemical and pharmacological profile for use as an in vitro PD model [49].

We evaluated the potential of the NB69 human neuroblastoma cell line to serve as an in vitro model of dopamine (DA) neurons for PD research. We compared it with differentiated NB69 cells. We differentiated the NB69 neuroblastoma cell line by adding dbcAMP to the culture medium, which induces neuronal differentiation and neurite growth (neuritogenesis/neurite inducer) [44]. This compound has previously been used and tested as a neural differentiator for various neural stem cells and cell lines [50,51]. We observed morphological and biochemical changes in NB69 cells treated with dbcAMP. These changes included the development of neuritic processes and an increased number of TH-positive cells. These results suggest that the compound induced NB69 cells to differentiate into a dopaminergic-like phenotype. These changes are consistent with those observed in the analyses of Mena et al., 1995 [44], which included studies of TH enzyme activity and decreased cell proliferation. These results confirm the change to a mature dopaminergic neuron.

In experimental PD research, the use of neurotoxic models, both in vitro and in vivo, is common. These models attempt to reproduce certain characteristics of the disease. They are very useful for understanding the causes and origins of the disease, as well as for searching for potential treatments, since there is no effective drug that can halt its progression. Methylphenylpyridinium (MPP+) is a neurotoxin that induces oxidative stress and is used as an in vitro PD model [16,17]. The effect on viability was analyzed in the NB69 neuroblastoma cell line and its differentiated counterparts, as well as in DMSCs. As expected, viability decreased in both phenotypes because the neurotoxic effects of MPP+ have been extensively studied and confirmed [13]. The toxic effect was greater in undifferentiated NB69 cells than in differentiated NB69 cells. This may be because differentiation produces a greater number of mitochondria, making cells less susceptible to MPP+ toxicity [52].

Because there are currently no effective therapies for PD, there is great anticipation surrounding the use of cell therapy for this condition. Mesenchymal stem cells (MSCs) appear to be a promising alternative for modifying PD progression because they provide neuroprotection, reduce neuroinflammation, and potentially repair damaged dopaminergic pathways [53]. The most commonly used MSCs are those derived from the umbilical cord (UC-MSCs) or adipose tissue (AD-MSCs). These cells have been shown to primarily act through paracrine signaling by secreting neurotrophic factors and exosomes. This promotes neuronal survival and modulates the microenvironment, mitigating oxidative stress [54]. In this study, we used decidua mesenchymal stromal cells (DMSCs). To our knowledge, no studies have examined the potential use of these stem cells in treating PD. Before examining the impact of DMSCs on MPP+-related toxicity in the in vitro PD model presented here, we assessed the influence of MPP+ on DMSC viability. MPP+ also affected viability, though to a much lesser degree than in NB69 cells. This effect could be mediated by organic cation transporter 3 (OCT3), which is expressed in numerous tissues, particularly in the placenta [55], including the decidua and the trophoblast [56]. In the placenta, OCT3 has been described as playing an important role in transporting cationic compounds, such as MPP+, from the fetal circulation to the placenta [57]. Additionally, the DA-1 dopamine receptor is present in the decidua of term placentas, possibly due to dopamine’s role in regulating prostaglandin production [58]. MSCs obtained from the basal decidua have also been shown to be resistant to toxic environments, suggesting their potential usefulness in treating diseases associated with oxidative and other types of stress [59].

We investigated the potential therapeutic effects of DMSCs in an MPP+-induced neurotoxicity model using dopaminergic NB69 cells and their differentiated counterparts. Previously, we observed that DMSCs possess a significant antitumor effect in both in vitro and in vivo models [33,42]. Therefore, we first analyzed the effects of DMSCs on NB69 cells in their two phenotypes: undifferentiated and differentiated. Results from co-culturing NB69 cells with DMSCs demonstrate that DMSCs negatively affect the viability of the undifferentiated NB69 phenotype. However, DMSCs co-cultured with differentiated NB69 cells did not demonstrate the expected antitumor effect; rather, they showed a slight increase in viability. In conclusion, the NB69 tumor cell line is not the most suitable method for studying the effect of DMSCs as a cell therapy for Parkinson’s disease. Instead, a model based on the differentiated NB69 cell line should be used [60].

A study was conducted to examine the therapeutic effects of DMSCs on MPP+-induced toxicity in NB69 cells. The results showed that DMSCs did not impact MPP+-induced damage or protect NB69 cells. This could be explained by the previously demonstrated antitumor properties of DMSC. However, DMSCs demonstrated a therapeutic effect in the MPP+ toxicity model in differentiated NB69 cells. DMSCs increased viability and significantly protected against cell damage. Some studies have shown that MSCs can exert a neuroprotective effect against oxidative stress by secreting neurotrophic factors (NTFs) including brain-derived neurotrophic factor (BDNF), nerve growth factor (NGF), and glial cell-derived neurotrophic factor (GDNF) [34,61]. Conversely, it has been established that decidua cells exhibit resistance to oxidative stress when exposed to elevated levels during pregnancy [62]. Considering these findings, it is reasonable to conclude that DMSCs can exert their therapeutic effects in environments with high oxidative stress, such as that generated by MPP+ treatment. This phenomenon could be attributed to the paracrine effect of DMSCs, resulting in the release of NTFs.

MPTP (1-methyl-4-phenyl-1,2,3,6-tetrahydropyridine) administration is a widely used in vivo model for PD. It is important to determine if DMSCs can be administered intravenously or via local transplantation for future in vivo neurotoxin studies. Therefore, in vitro migration assays were used to analyze whether neuronal damage attracts DMSCs. The results showed that DMSCs were significantly attracted to undifferentiated NB69 cells, regardless of whether the NB69 cells had been damaged by MPP+. This may be due to the tumorigenic nature of the NB69 cells, which is consistent with previous research conducted in our laboratory demonstrating DMSCs’ attraction to tumorous breast tissue [33,42]. Interestingly, the results showed that DMSCs were only significantly attracted to differentiated NB69 cells when those cells were damaged by MPP+. These results align with the observation that MSCs migrate to areas of tissue damage and suggest that DMSCs can be administered intravenously [33].

MPP+ is one of the most widely used neurotoxins in in vitro PD models because it specifically targets dopaminergic neurons and induces their death by inhibiting complex I of the mitochondrial respiratory chain [15]. This model simulates some of the crucial pathological features of PD, including mitochondrial dysfunction, oxidative stress, and apoptosis [63]. MitoSOX™ Red allows for the direct evaluation of mitochondrial superoxide anion (O_2_•–) production in live cells. Under basal conditions, most cell types exhibit low to moderate red fluorescence, corresponding to physiological levels of superoxide generated by the respiratory chain [64]. Significant increases in MitoSOX fluorescence are observed under conditions that cause mitochondrial dysfunction or increased electron flow. Examples include inhibiting complexes I or III (e.g., rotenone or antimycin A) or metabolic overload [65]. The results presented here suggest that DMSCs protect mitochondrial function against damage caused by the neurotoxin MPP+ in differentiated NB69 cells. This may be one of the mechanisms by which DMSCs exert a regenerative effect in PD. However, this study is still in the preliminary stages and is based on in vitro research. Further preclinical research in vivo is needed to determine the true role of DMSCs in PD. Mitochondrial dysfunction significantly contributes to the progression of various diseases, making it a key therapeutic target. Preclinical evidence indicates that MSCs can enhance mitochondrial function in damaged tissues and exert therapeutic effects in various pathologies, including neurological diseases [66]. It has been suggested that MSCs regulate mitochondrial biogenesis, mitophagy, and mitochondrial fission/fusion [67]. Mitochondria derived from UC-MSCs reduce the death of DA neurons caused by MPP+ and other toxins [68]. Future studies should investigate whether DMSCs transfer mitochondria to NB69 cells to protect them from MPP+-induced mitochondrial damage.

## 4. Materials and Methods

### 4.1. Isolation, Culture, and Characterization of Decidua Mesenchymal Stromal Cells (DMSCs)

Human placentas were collected from healthy mothers at the Department of Obstetrics and Gynecology during vaginal or cesarean deliveries. Collection was approved by the Ethics Committee of Hospital Universitario 12 de Octubre and followed written informed consent. Placental membranes were dissected, and cells were obtained as previously described [36]. Briefly, the tissue was digested twice with trypsin (Gibco, Thermo Fisher Scientific, Waltham, MA, USA) for 30 min. The isolated cells were cultured at 37 °C, 5% CO_2_ and 95% humidity in Dulbecco’s modified Eagle’s medium (DMEM) supplemented with 2 mM of glutamax, 0.1 mM of sodium pyruvate (Gibco, Thermo Fisher Scientific, Waltham, MA, USA), 1% nonessential amino acids (NEAA) (STEMCELL Technologies, Vancouver, BC, Canada), 1% penicillin/streptomycin (Gibco, Thermo Fisher Scientific, Waltham, MA, USA), 10% of fetal bovine serum (FBS) (Biowest, Nuaillé, France) and 10 ng/mL of Epidermal Growth Factor (EGF) (Sigma-Aldrich, St. Louis, MO, USA).

DMSCs isolated from five different placentas were used in this study. DMSCs from passages 3 to 5 were characterized by flow cytometry using antibodies against CD105-PE-Cy7, CD90-APC, CD73-SB600, CD44-AF488, CD45-PerCP-Cy5.5, and CD34-PE (Invitrogen, Thermo Fisher Scientific, Waltham, MA, USA). Cell fluorescence was assessed using a Cytek Aurora spectral flow cytometer (Cytek Biosciences, Fremont, CA, USA). The percentage of positive cells was determined by analyzing the data using Flore-ada.io software (https://floreada.io/). The results demonstrate that DMSCs exhibit an MSC phenotype, as evidenced by their expression of CD105, CD90, CD73, and CD44, and their lack of CD45 and CD34 expression (Supplementary Figure S1). A representative result from the five experiments analyzed is shown.

### 4.2. Cell Culture and Differentiation of Neuroblastoma Cell Line NB69

The human neuroblastoma cell line NB69 was kindly provided by Dr. Maria Angeles Mena [49]. The NB69 cells were grown in DMEM supplemented with 2 mM of glutamax, 0.1 mM of sodium pyruvate, 1% NEAA, 1% penicillin/streptomycin, and 10% of FBS at 37 °C in a humidified atmosphere with 5% CO_2_.

To induce differentiation, NB69 cells were seeded at an initial concentration of 1 × 10^4^ cells/cm^2^ After 48 h, differentiation was induced by adding 2 mM dbcAMP (STEMCELL Technologies, Vancouver, BC, Canada) to the culture medium, which had been supplemented with 2% FBS. This was maintained for five days, according to the published procedure by Mena, M.A. et al., 1989 [49]. The differentiation stage of NB69 cells was assessed by three approaches: an immunofluorescence assay to detect tyrosine hydroxylase (TH) protein expression, measurement of neurite size, and quantification of the cell proliferation rate (see below). Once differentiation was complete, MPP+ neurotoxin was added to the differentiated NB69 cells at a concentration of 0.5 mM. The toxin was allowed to act for 6 h before establishing the co-culture with DMSC, as described in Section 4.7.

### 4.3. Preparation of MPP+ Toxin and Treatment of NB69 Cells

1-Methyl-4-phenylpyridinium iodide (MPP+) (Thermo Fisher Scientific, Waltham, MA, USA) was freshly prepared in phosphate-buffered saline (PBS) prior to use. Different doses and incubation times with the MPP+ toxin were tested in this study (see Section 2).

### 4.4. Cell Viability Assay

Cell viability after MPP+ treatment was quantified using the Alamar Blue assay (Invitrogen, Thermo Fisher Scientific, Madrid, Spain), according to the manufacturer’s instructions. Specifically, 25,000 cells/cm^2^ were seeded and treated with MPP+ at various times, as indicated in the figure captions. The culture medium was then replaced with complete DMEM containing 1% Alamar Blue reagent, and the cells were incubated for 1 h at 37 °C to allow viable cells to convert resazurin (blue) to resorufin (violet). The fluorescence signal was measured at 590 nm using a multimodal plate reader (2300 Enspire, PerkinElmer España S.L., Madrid, Spain).

### 4.5. Cell Death Assay

MPP+-induced cytotoxicity was determined by measuring the release of lactate dehydrogenase (LDH) from damaged NB69 cells into the culture medium. This was done using the Pierce LDH Cytotoxicity Assay Kit (Thermo Scientific, Madrid, Spain), according to the manufacturer’s instructions. Briefly, after treatment, the cell culture medium was collected, a reaction mixture was added at a 1:1 ratio, and the samples were incubated for 30 min at room temperature, protected from light. The reaction was then stopped, and the absorbance was quantified at 460 nm and 680 nm. The difference between the absorbance value at 680 nm (instrument background signal) and the absorbance value at 490 nm indicates LDH activity. Annexin V and propidium iodide (PI) labeling was performed on cells in suspension, in darkness, and at room temperature. After 15 min of incubation, the labeled cells were analyzed by flow cytometry (FACScalibur, BD Biosciences, San Jose, CA, USA).

### 4.6. Cell Proliferation Assay

Cell proliferation in NB69 cells was quantified using the Cell Proliferation Colorimetric ELISA (Roche Diagnostics, S.L., Sant Cugat del Vallès, Barcelona, Spain) by measuring the incorporation of 5-bromo-2-deoxyuridine (BrdU) during DNA synthesis in dividing cells. Incorporated BrdU was detected using a colorimetric immunoassay according to the manufacturer’s instructions. Briefly, the BrdU labeling solution was added directly to the cell culture and incubated for 24 h. The cells were fixed, and the BrdU-labeled DNA was denatured to make BrdU accessible to the anti-BrdU-POD antibody. Then, the substrate solution was added, and absorbance was measured at 370 nm and 492 nm using an Enspire 2300 Multimodal Plate Reader (PerkinElmer España S.L., Madrid, Spain).

### 4.7. Indirect Co-Culture Assay of NB69 and DMSCs

To study the effect of DMSCs on NB69 cells and differentiated NB69 cells, an indirect co-culture assay was established using Transwell inserts (Corning Costar, Corning Incorporated, Corning, NY, USA) containing a polycarbonate membrane with a pore size of 0.4 μm. This Transwell-based co-culture system provides an indirect in vitro environment in which the DMSCs and NB69 cells are separated by a permeable membrane that permits paracrine signaling without direct cell–cell contact. NB69 cells were seeded at a concentration of 1 × 10^5^ cells per 24-well plate. DMSCs were seeded at ratios of 1:2, 1:5 or 1:10 to NB69 cells on the Transwell inserts. Both cell lines were cultured separately for 24 h to allow them to form a cell monolayer. Co-culture was established by placing the DMSC-Transwells onto NB69 wells. For differentiated NB69 cells, the co-culture was established after differentiation was complete. Before establishing the co-culture with DMSC, MPP+ neurotoxin was added to the differentiated NB69 at a concentration of 0.5 mM to allow the toxin to take effect for 6 h before proceeding with the co-culture. Viability was assessed using the Alamar Blue assay 72 h after establishing the co-culture with DMSCs, as described above.

### 4.8. DMSC Migration Assay

DMSCs were seeded in Transwell inserts with an 8 μm pore size polycarbonate membrane, and NB69 cells were seeded in the plate wells. The NB69 cells were seeded at a concentration of 1 × 10^4^ cells/cm^2^ in the culture plate. The DMSCs were seeded at a 1:5 ratio (DMSC:NB69) in the Transwell inserts. Five different chemoattractant conditions were placed in the bottom chambers: DMEM (negative control), NB69 cells, NB69 cells treated with 0.5 mM MPP+, differentiated NB69 cells, and differentiated NB69 cells treated with 0.5 mM MPP+. The cells were co-cultured for 3 h, after which DMSC migration was studied using the CytoSelect Cell Migration Assay kit (Cell Biolabs, Bionova Cientifica, S.L., Madrid, Spain) [33]. Non-migratory cells were removed from the top of the Transwell membrane, the migratory cells were stained, and once the color was extracted, the absorbance was quantified at 560 nm using an Enspire 2300 plate reader (PerkinElmer España S.L., Madrid, Spain).

### 4.9. Immunofluorescence

To identify dopamine-secreting neurons, tyrosine hydroxylase (TH) immunoreactivity was studied using immunofluorescence. Differentiated NB69 cells were fixed in 4% paraformaldehyde for 15 min, permeabilized with 0.3% Triton X-100 for 10 min, and blocked with 1% BSA in PBS for 30 min. The cells were then incubated overnight at 4 °C with a rabbit polyclonal anti-TH antibody (1:250) (Merck Millipore, Madrid, Spain) and for 1 h at room temperature with a 1:200 dilution of a secondary anti-FITC antibody (Santa Cruz Biotechnology, Dallas, TX, USA) diluted in PBS with 1% BSA. Nuclei were stained with 0.1 ng/mL of 4′,6-diamidino-2-phenylindole (DAPI) (Sigma-Aldrich, St. Louis, MO, USA) for 15 min. Fluorescence was visualized using a Leica DMIL microscope equipped with a DC.3000S SCMEX 3 camera (Leica Microsystems, Wetzlar, Germany), and images were acquired using ImageFocus v4 software (Euromex Microscopen, BV, Arnhem, TBV, Arnhem, The Netherlands). Total cell and background fluorescence intensities were quantified using ImageJ (National Institute of Mental Health, Bethesda, MD, USA, version 1.54g). Corrected total cell fluorescence (CTCF) was calculated using the formula: CTCF = Integrated density − (Selected cell area × Mean fluorescence of background readings).

### 4.10. Measurement of Neurite Length

Using ImageJ software, neurites and somas were delineated and measured from images of undifferentiated and differentiated NB69 cells. The software automatically calculates the length of the plotted segments and exports the data. Axon and soma length data were analyzed to obtain the axon/soma ratio.

### 4.11. Detection of Mitochondrial Reactive Oxygen Species (ROS) Levels

Mitochondrial superoxide anion levels were measured using the fluorogenic dye MitoSOX (Invitrogen, Thermo Fisher Scientific, Waltham, MA, USA). The NB69 medium was first removed, and then the cells were washed twice with Hank’s Balanced Salt Solution (HBSS) (Gibco, Thermo Fisher Scientific, Waltham, MA, USA). Then, 5 µM of MitoSOX diluted in HBSS was added to each well. The cells were incubated at 37 °C in a humidified environment for 30 min. The culture was then rinsed twice with HBSS to facilitate inspection with a fluorescence microscope.

Live cells were observed using a fluorescence microscope (ZOE Fluorescent Cell Imager, Bio-Rad Laboratories, Hercules, CA, USA) for undifferentiated NB69 cells and a Leica Dmi8 inverted microscope (Leica Microsystems, Wetzlar, Germany) for differentiated NB69 cells.

Fluorescence intensity was quantified using ImageJ software. Next, the mean fluorescence intensity (MFI) of each positive cell (i.e., the region of interest [ROI]) was measured. Five representative images were randomly captured for each condition, and the mean of all regions of interest (ROIs) was calculated as the data for that particular well.

After fluorescence microscopy imaging, one study detached differentiated NB69 cells from their well and analyzed them using a Cytek Aurora spectral flow cytometer (Cytek Biosciences, Fremont, CA, USA) to determine the percentage of Mito-SOX-positive cells. The data were analyzed using Floreada.io software.

### 4.12. Statistical Analysis

The normality of data was assessed using the Shapiro–Wilk or Kolmogorov–Smirnov tests in the graphPad 8.0 program. Based on these results, a one-way ANOVA was performed in cases of homogeneity of variance, followed by a Bonferroni post hoc test for multiple comparisons between experimental groups. Pairwise comparisons were analyzed using Student’s *t*-test or with Mann-Whitney U-test. All results are expressed as mean ± standard deviation (SD). Results were considered statistically significant when *p* < 0.05.

## 5. Conclusions

This study revealed key aspects of in vitro Parkinson’s disease modeling. Although the neurotoxic effects of MPP+ are well-known, its impact on other cell types requires further verification. Even though the neuroblastoma cell line is commonly used to study PD, our results demonstrate that it is not ideal for all studies. In fact, this study demonstrates that the neuroblastoma cell line model is not suitable for studying cell therapy with DMSCs due to their antitumor effect. Using dbcAMP-differentiated NB69 cells revealed that DMSCs have a significant neuroprotective effect against MPP+-induced cell damage in this more neuronal and dopaminergic cell type. This neuroprotective effect is due, at least in part, to improved mitochondrial function, a mechanism that should be studied further. Taken together, our results highlight the potential relevance of human DMSCs for future translational research aimed at developing cell-based therapies for Parkinson’s disease and other neurodegenerative conditions.

## Data Availability

The data supporting the conclusions of this article will be made available to interested researchers upon request to the authors.

## Supplementary Materials

The following supporting information can be downloaded at: https://www.mdpi.com/article/10.3390/ijms27093925/s1.

## References

[1] Connolly B.S., Lang A.E. (2014). Pharmacological treatment of Parkinson disease: A review. JAMA.

[2] Wirdefeldt K., Adami H.O., Cole P., Trichopoulos D., Mandel J. (2011). Epidemiology and etiology of Parkinson’s disease: A review of the evidence. Eur. J. Epidemiol..

[3] Marti M.J., Tolosa E., Campdelacreu J. (2003). Clinical overview of the synucleinopathies. Mov. Disord..

[4] Rcom-H’cheo-Gauthier A., Goodwin J., Pountney D.L. (2014). Interactions between calcium and alpha-synuclein in neurodegeneration. Biomolecules.

[5] Chaudhuri K.R., Schapira A.H. (2009). Non-motor symptoms of Parkinson’s disease: Dopaminergic pathophysiology and treatment. Lancet Neurol..

[6] Dexter D.T., Jenner P. (2013). Parkinson disease: From pathology to molecular disease mechanisms. Free Radic. Biol. Med..

[7] Mensikova K., Matej R., Colosimo C., Rosales R., Tuckova L., Ehrmann J., Hrabos D., Kolarikova K., Vodicka R., Vrtel R. (2022). Lewy body disease or diseases with Lewy bodies?. NPJ Park. Dis..

[8] Perez R.G., Hastings T.G. (2004). Could a loss of alpha-synuclein function put dopaminergic neurons at risk?. J. Neurochem..

[9] Franco-Iborra S., Vila M., Perier C. (2016). The Parkinson Disease Mitochondrial Hypothesis: Where Are We at?. Neuroscientist.

[10] Stolzenberg E., Berry D., Yang D., Lee E.Y., Kroemer A., Kaufman S., Wong G.C.L., Oppenheim J.J., Sen S., Fishbein T. (2017). A Role for Neuronal Alpha-Synuclein in Gastrointestinal Immunity. J. Innate Immun..

[11] Pajares M., Rojo A.I., Manda G., Bosca L., Cuadrado A. (2020). Inflammation in Parkinson’s Disease: Mechanisms and Therapeutic Implications. Cells.

[12] Gao H.L., Li C., Nabeka H., Shimokawa T., Saito S., Wang Z.Y., Cao Y.M., Matsuda S. (2013). Attenuation of MPTP/MPP(+) toxicity in vivo and in vitro by an 18-mer peptide derived from prosaposin. Neuroscience.

[13] Singer T.P., Ramsay R.R. (1990). Mechanism of the neurotoxicity of MPTP. An update. FEBS Lett..

[14] Liu M., Zuo S., Guo X., Peng J., Xing Y., Guo Y., Li C., Xing H. (2023). The Study of Overexpression of Peroxiredoxin-2 Reduces MPP(+)-Induced Toxicity in the Cell Model of Parkinson’s Disease. Neurochem. Res..

[15] Risiglione P., Leggio L., Cubisino S.A.M., Reina S., Paterno G., Marchetti B., Magri A., Iraci N., Messina A. (2020). High-Resolution Respirometry Reveals MPP(+) Mitochondrial Toxicity Mechanism in a Cellular Model of Parkinson’s Disease. Int. J. Mol. Sci..

[16] Dong G., Fan L., Li C., Jiao Y., Li X., Li H., Liang Y., Ren Y., Wang L., Xiao D. (2025). Schisanhenol Inhibits MPTP/MPP(+)-Induced Ferroptosis in Dopaminergic Neurons Via Nrf2/TrxR1/GPX4 Pathway against Parkinson’s Disease. Neurochem. Res..

[17] Svay T., Pariyar R., Bastola T., Yoon C.S., Lee E.S., Oh H., Seo J. (2026). 1beta,6alpha-Dihydroxyeudesm-4(15)-ene Protects Against MPP(+)-Induced Cytotoxicity in SH-SY5Y Cells: An In Vitro Model of Parkinson’s Disease. J. Med. Food.

[18] Nakamura K., Bindokas V.P., Marks J.D., Wright D.A., Frim D.M., Miller R.J., Kang U.J. (2000). The selective toxicity of 1-methyl-4-phenylpyridinium to dopaminergic neurons: The role of mitochondrial complex I and reactive oxygen species revisited. Mol. Pharmacol..

[19] Perfeito R., Cunha-Oliveira T., Rego A.C. (2013). Reprint of: Revisiting oxidative stress and mitochondrial dysfunction in the pathogenesis of Parkinson disease-resemblance to the effect of amphetamine drugs of abuse. Free Radic. Biol. Med..

[20] Kim T.Y., Lee B.D. (2025). Current therapeutic strategies in Parkinson’s disease: Future perspectives. Mol. Cells.

[21] Seppi K., Ray Chaudhuri K., Coelho M., Fox S.H., Katzenschlager R., Perez Lloret S., Weintraub D., Sampaio C., the collaborators of the Parkinson’s Disease Update on Non-Motor Symptoms Study Group on behalf of the Movement Disorders Society Evidence-Based Medicine Committee (2019). Update on treatments for nonmotor symptoms of Parkinson’s disease-an evidence-based medicine review. Mov. Disord..

[22] Zahoor I., Shafi A., Haq E., Stoker T.B., Greenland J.C. (2018). Pharmacological Treatment of Parkinson’s Disease. Parkinson’s Disease: Pathogenesis and Clinical Aspects.

[23] Warren Olanow C., Kieburtz K., Rascol O., Poewe W., Schapira A.H., Emre M., Nissinen H., Leinonen M., Stocchi F., Stalevo Reduction in Dyskinesia Evaluation in Parkinson’s Disease (STRIDE-PD) Investigators (2013). Factors predictive of the development of Levodopa-induced dyskinesia and wearing-off in Parkinson’s disease. Mov. Disord..

[24] Liu Z., Cheung H.H. (2020). Stem Cell-Based Therapies for Parkinson Disease. Int. J. Mol. Sci..

[25] Zhang A.L., Wen L. (2026). Synergetic pathways for Parkinson’s disease therapy: The intersection of exercise and stem cell science. World J. Stem Cells.

[26] Jang S.E., Qiu L., Chan L.L., Tan E.K., Zeng L. (2020). Current Status of Stem Cell-Derived Therapies for Parkinson’s Disease: From Cell Assessment and Imaging Modalities to Clinical Trials. Front. Neurosci..

[27] Lindvall O. (2016). Clinical translation of stem cell transplantation in Parkinson’s disease. J. Intern. Med..

[28] Piccini P., Pavese N., Hagell P., Reimer J., Bjorklund A., Oertel W.H., Quinn N.P., Brooks D.J., Lindvall O. (2005). Factors affecting the clinical outcome after neural transplantation in Parkinson’s disease. Brain.

[29] Clark B.J., Lelos M.J., Loring J.F. (2024). Advancing Parkinson’s disease treatment: Cell replacement therapy with neurons derived from pluripotent stem cells. Stem Cells.

[30] Politis M., Lindvall O. (2012). Clinical application of stem cell therapy in Parkinson’s disease. BMC Med..

[31] Thanaskody K., Jusop A.S., Tye G.J., Wan Kamarul Zaman W.S., Dass S.A., Nordin F. (2022). MSCs vs. iPSCs: Potential in therapeutic applications. Front. Cell Dev. Biol..

[32] Liu L., Eckert M.A., Riazifar H., Kang D.K., Agalliu D., Zhao W. (2013). From blood to the brain: Can systemically transplanted mesenchymal stem cells cross the blood-brain barrier?. Stem Cells Int..

[33] Vegh I., Grau M., Gracia M., Grande J., de la Torre P., Flores A.I. (2013). Decidua mesenchymal stem cells migrated toward mammary tumors in vitro and in vivo affecting tumor growth and tumor development. Cancer Gene Ther..

[34] Volkman R., Offen D. (2017). Concise Review: Mesenchymal Stem Cells in Neurodegenerative Diseases. Stem Cells.

[35] Torre P., Flores A.I. (2020). Current Status and Future Prospects of Perinatal Stem Cells. Genes.

[36] Macias M.I., Grande J., Moreno A., Dominguez I., Bornstein R., Flores A.I. (2010). Isolation and characterization of true mesenchymal stem cells derived from human term decidua capable of multilineage differentiation into all 3 embryonic layers. Am. J. Obstet. Gynecol..

[37] Bravo B., Gallego M.I., Flores A.I., Bornstein R., Puente-Bedia A., Hernandez J., de la Torre P., Garcia-Zaragoza E., Perez-Tavarez R., Grande J. (2016). Restrained Th17 response and myeloid cell infiltration into the central nervous system by human decidua-derived mesenchymal stem cells during experimental autoimmune encephalomyelitis. Stem Cell Res. Ther..

[38] de la Torre P., Paris J.L., Fernandez-de la Torre M., Vallet-Regi M., Flores A.I. (2021). Endostatin Genetically Engineered Placental Mesenchymal Stromal Cells Carrying Doxorubicin-Loaded Mesoporous Silica Nanoparticles for Combined Chemo- and Antiangiogenic Therapy. Pharmaceutics.

[39] De La Torre P., Perez-Lorenzo M.J., Alcazar-Garrido A., Collado J., Martinez-Lopez M., Forcen L., Masero-Casasola A.R., Garcia A., Gutierrez-Velez M.C., Medina-Polo J. (2022). Perinatal mesenchymal stromal cells of the human decidua restore continence in rats with stress urinary incontinence induced by simulated birth trauma and regulate senescence of fibroblasts from women with stress urinary incontinence. Front. Cell Dev. Biol..

[40] Paris J.L., de la Torre P., Cabanas M.V., Manzano M., Flores A.I., Vallet-Regi M. (2019). Suicide-gene transfection of tumor-tropic placental stem cells employing ultrasound-responsive nanoparticles. Acta Biomater..

[41] Paris J.L., de la Torre P., Flores A.I. (2021). New Therapeutic Approaches for Allergy: A Review of Cell Therapy and Bio- or Nano-Material-Based Strategies. Pharmaceutics.

[42] Paris J.L., de la Torre P., Manzano M., Cabanas M.V., Flores A.I., Vallet-Regi M. (2016). Decidua-derived mesenchymal stem cells as carriers of mesoporous silica nanoparticles. In vitro and in vivo evaluation on mammary tumors. Acta Biomater..

[43] Paris J.L., de la Torre P., Victoria Cabanas M., Manzano M., Grau M., Flores A.I., Vallet-Regi M. (2017). Vectorization of ultrasound-responsive nanoparticles in placental mesenchymal stem cells for cancer therapy. Nanoscale.

[44] Mena M.A., Casarejos M.J., Bonin A., Ramos J.A., Garcia Yebenes J. (1995). Effects of dibutyryl cyclic AMP and retinoic acid on the differentiation of dopamine neurons: Prevention of cell death by dibutyryl cyclic AMP. J. Neurochem..

[45] Lopes F.M., Bristot I.J., da Motta L.L., Parsons R.B., Klamt F. (2017). Mimicking Parkinson’s Disease in a Dish: Merits and Pitfalls of the Most Commonly used Dopaminergic In Vitro Models. Neuromol. Med..

[46] Ferrari E., Cardinale A., Picconi B., Gardoni F. (2020). From cell lines to pluripotent stem cells for modelling Parkinson’s Disease. J. Neurosci. Methods.

[47] Mena M.A., Pardo B., Casarejos M.J., Fahn S., Garcia de Yebenes J. (1992). Neurotoxicity of levodopa on catecholamine-rich neurons. Mov. Disord..

[48] Rodriguez-Martin E., Canals S., Casarejos M.J., de Bernardo S., Handler A., Mena M.A. (2001). L-DOPA and glia-conditioned medium have additive effects on tyrosine hydroxylase expression in human catecholamine-rich neuroblastoma NB69 cells. J. Neurochem..

[49] Mena M.A., Garcia de Yebenes J., Dwork A., Fahn S., Latov N., Herbert J., Flaster E., Slonim D. (1989). Biochemical properties of monoamine-rich human neuroblastoma cells. Brain Res..

[50] Harberts J., Siegmund M., Schnelle M., Zhang T., Lei Y., Yu L., Zierold R., Blick R.H. (2021). Robust neuronal differentiation of human iPSC-derived neural progenitor cells cultured on densely-spaced spiky silicon nanowire arrays. Sci. Rep..

[51] Kim H., Zahir T., Tator C.H., Shoichet M.S. (2011). Effects of dibutyryl cyclic-AMP on survival and neuronal differentiation of neural stem/progenitor cells transplanted into spinal cord injured rats. PLoS ONE.

[52] Khwanraj K., Phruksaniyom C., Madlah S., Dharmasaroja P. (2015). Differential Expression of Tyrosine Hydroxylase Protein and Apoptosis-Related Genes in Differentiated and Undifferentiated SH-SY5Y Neuroblastoma Cells Treated with MPP(.). Neurol. Res. Int..

[53] Tambe P., Undale V., Sanap A., Bhonde R., Mante N. (2024). The prospective role of mesenchymal stem cells in Parkinson’s disease. Park. Relat. Disord..

[54] Tew V.K., Barathan M., Nordin F., Law J.X., Ng M.H. (2025). Emerging Role of Mesenchymal Stromal Cell and Exosome Therapies in Treating Cognitive Impairment. Pharmaceutics.

[55] Lee W.K., Wolff N.A., Thevenod F. (2009). Organic cation transporters: Physiology, toxicology and special focus on ethidium as a novel substrate. Curr. Drug Metab..

[56] Uhlen M., Fagerberg L., Hallstrom B.M., Lindskog C., Oksvold P., Mardinoglu A., Sivertsson A., Kampf C., Sjostedt E., Asplund A. (2015). Proteomics. Tissue-based map of the human proteome. Science.

[57] Sata R., Ohtani H., Tsujimoto M., Murakami H., Koyabu N., Nakamura T., Uchiumi T., Kuwano M., Nagata H., Tsukimori K. (2005). Functional analysis of organic cation transporter 3 expressed in human placenta. J. Pharmacol. Exp. Ther..

[58] Yanagawa T., Kishimoto Y., Tada K., Arai F., Kondo Y., Kudo T. (1997). Presence of dopamine DA-1 receptors in human decidua. Placenta.

[59] Khatlani T., Algudiri D., Alenzi R., Al Subayyil A.M., Abomaray F.M., Bahattab E., AlAskar A.S., Kalionis B., El-Muzaini M.F., Abumaree M.H. (2018). Preconditioning by Hydrogen Peroxide Enhances Multiple Properties of Human Decidua Basalis Mesenchymal Stem/Multipotent Stromal Cells. Stem Cells Int..

[60] Carvajal-Oliveros A., Roman-Martinez C., Reynaud E., Martinez-Martinez E. (2024). The BE (2)-M17 neuroblastoma cell line: Revealing its potential as a cellular model for Parkinson’s disease. Front. Cell. Neurosci..

[61] Yari H., Mikhailova M.V., Mardasi M., Jafarzadehgharehziaaddin M., Shahrokh S., Thangavelu L., Ahmadi H., Shomali N., Yaghoubi Y., Zamani M. (2022). Emerging role of mesenchymal stromal cells (MSCs)-derived exosome in neurodegeneration-associated conditions: A groundbreaking cell-free approach. Stem Cell Res. Ther..

[62] Abomaray F.M., Al Jumah M.A., Alsaad K.O., Jawdat D., Al Khaldi A., AlAskar A.S., Al Harthy S., Al Subayyil A.M., Khatlani T., Alawad A.O. (2016). Phenotypic and Functional Characterization of Mesenchymal Stem/Multipotent Stromal Cells from Decidua Basalis of Human Term Placenta. Stem Cells Int..

[63] Angelova P.R., Barilani M., Lovejoy C., Dossena M., Vigano M., Seresini A., Piga D., Gandhi S., Pezzoli G., Abramov A.Y. (2018). Mitochondrial dysfunction in Parkinsonian mesenchymal stem cells impairs differentiation. Redox Biol..

[64] Mukhopadhyay P., Rajesh M., Yoshihiro K., Hasko G., Pacher P. (2007). Simple quantitative detection of mitochondrial superoxide production in live cells. Biochem. Biophys. Res. Commun..

[65] Dikalov S.I., Harrison D.G. (2014). Methods for detection of mitochondrial and cellular reactive oxygen species. Antioxid. Redox Signal..

[66] Chen H., Chen X., Zhou Z.H., Zheng J.R., Lu Y., Lin P., Lin Y.F., Zheng Y.C., Xiong B., Xu R.W. (2025). Mesenchymal stromal cell-mediated mitochondrial transfer unveils new frontiers in disease therapy. Stem Cell Res. Ther..

[67] Mukkala A.N., Jerkic M., Khan Z., Szaszi K., Kapus A., Rotstein O. (2023). Therapeutic Effects of Mesenchymal Stromal Cells Require Mitochondrial Transfer and Quality Control. Int. J. Mol. Sci..

[68] Eo H., Yu S.H., Choi Y., Kim Y., Kang Y.C., Lee H., Kim J.H., Han K., Lee H.K., Chang M.Y. (2024). Mitochondrial transplantation exhibits neuroprotective effects and improves behavioral deficits in an animal model of Parkinson’s disease. Neurotherapeutics.

