# Supplementary material for: Protective Effect of Placental Mesenchymal Stromal Cells in an In Vitro Model of Parkinson’s Disease Using Differentiated Neuroblastoma Cells
Source: Int J Mol Sci. 2026 Apr 28;27(9):3925. doi: 10.3390/ijms27093925 (PMC13163953; doi:10.3390/ijms27093925)
Supplement: Supplementary file 1 [file ijms-27-03925-s001.zip › ijms-4255256-supplementary.pdf]

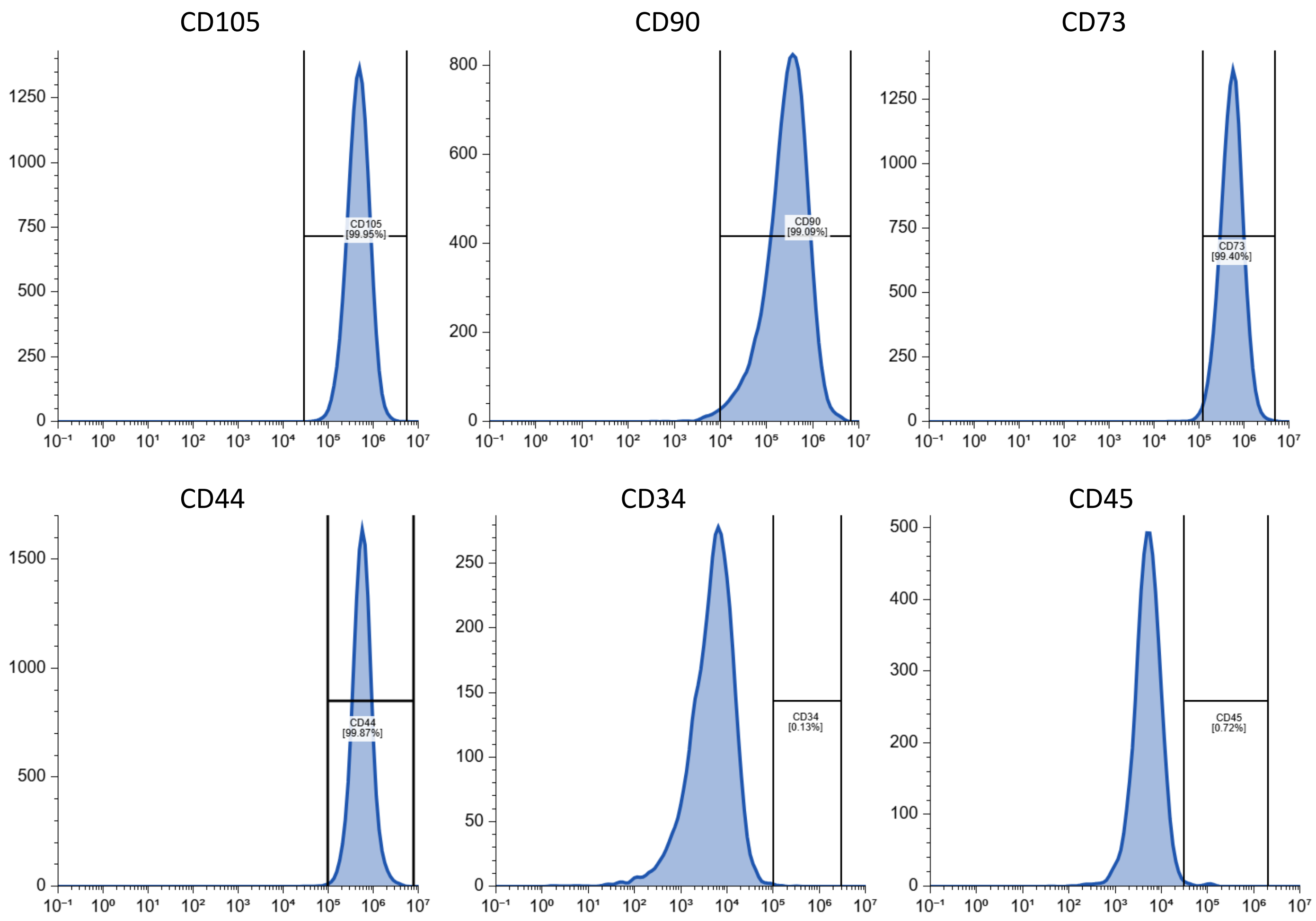

Supplementary Figure S1. DMSC immunophenotype by flow cytometry. DMSCs from passages 3 to 5 were analyzed by flow cytometry after staining with the specific antibodies. A representative example of five experiments is shown. Ninety-nine percent of the analyzed cells were positive for CD90, CD73, and CD44, and negative for CD45 and CD34. These results are consistent with the phenotype of mesenchymal stromal cells.
